# The DIR Gene Family in Watermelon: Evolution, Stress Expression Profiles, and Functional Exploration of *ClDIR8*

**DOI:** 10.3390/ijms26167730

**Published:** 2025-08-10

**Authors:** Kaijing Zhang, Zhu Wang, Huiyu Tian, Jiong Gao, Rongjing Cui, Yingjie Shu, Qiangqiang Ding, Li Jia, Congsheng Yan

**Affiliations:** 1 Key Laboratory of Horticultural Crop Germplasm Innovation and Utilization (Co-Construction by Ministry and Province), Institute of Horticulture, Anhui Academy of Agricultural Sciences, Hefei 230001, China; zhangkj@ahstu.edu.cn (K.Z.); qqdding@163.com (Q.D.); 2 College of Agriculture, Anhui Science and Technology University, Fengyang 233100, China; yjs2024287@ahstu.edu.cn (Z.W.); tianhuiyu1128@163.com (H.T.); yjs2023182@ahstu.edu.cn (J.G.); yjs2023176@ahstu.edu.cn (R.C.); shuyj@ahstu.edu.cn (Y.S.); 3 Anhui Provincial Key Laboratory for Germplasm Resources Creation and High-Efficiency Cultivation of Horticultural Crops, Hefei 230001, China; 4 Institute of Vegetables, Anhui Academy of Agricultural Sciences, Hefei 230001, China

**Keywords:** watermelon, DIR, gene family, bioinformatics, expression analysis, subcellular localization, interacting proteins

## Abstract

Dirigent proteins (DIR) are involved in lignan biosynthesis, stress responses, and disease resistance in plants. However, systematic characterization of the DIR gene family in watermelon (*Citrullus lanatus*) remains limited. Here, we identified 22 *ClDIR* genes in watermelon using bioinformatics methods, designated *ClDIR1* to *ClDIR22*, which were unevenly distributed across eight chromosomes and classified into three subfamilies (DIR-a, DIR-b/d, DIR-e) based on phylogenetic analysis, with DIR-b/d being the largest. Synteny analysis revealed that tandem duplication primarily drove *ClDIR* family expansion, and collinear relationships with *Arabidopsis*, rice, and cucurbit species indicated evolutionary conservation. *Cis*-acting element analysis showed abundant stress- and hormone-responsive elements in *ClDIR* promoters, suggesting roles in stress regulation. Tissue-specific expression analysis demonstrated distinct patterns, with most genes highly expressed in roots. Expression profiling under 16 abiotic and biotic stresses showed 18 *ClDIR* genes responded to stress, with *ClDIR8* differentially expressed across all conditions. qRT-PCR validation of six key genes (*ClDIR5*, *ClDIR8*, *ClDIR9*, *ClDIR12*, *ClDIR16*, *ClDIR22*) confirmed their expression patterns under high-temperature, drought, salt, and low-temperature stresses, showing a high degree of consistency with transcriptome data. Subcellular localization indicated ClDIR8 is peroxisome-localized. Yeast two-hybrid (Y2H) and bimolecular fluorescence complementation (BiFC) assays validated two ClDIR8-interacting proteins, Cla97C02G049920 (encoding peroxidase) and Cla97C08G152180 (encoding catalase). These findings provide insights into *ClDIR* genes in watermelon, highlighting *ClDIR8* as a key stress-responsive candidate for further functional studies and breeding.

## 1. Introduction

Plants continuously face dual challenges of abiotic and biotic stresses during growth and development. These environmental stressors disrupt cellular redox homeostasis, dismantle hormonal signaling networks, and inhibit the activity of photosynthetic electron transport chains [1,2,3], consequently leading to reduced photosynthetic carbon assimilation rates, imbalanced source–sink transport of assimilates, and blocked secondary metabolite biosynthesis. This ultimately results in crop yield loss and quality degradation. Due to their sessile growth habit, plants cannot evade environmental pressures through physical displacement and must rely on physical and chemical strategies to counteract biotic and abiotic stresses [4]. For instance, plants enhance cell wall rigidity by synthesizing lignin to resist environmental pressures [5]. Notably, lignin biosynthesis is closely associated with DIR (dirigent) proteins, which are encoded by the DIR gene family and specifically regulate the stereoselectivity of lignin monomer coupling, a key step in secondary cell wall thickening [6]. Additionally, these proteins dynamically regulate lignin deposition in cell walls in response to environmental signals [7,8]. Thus, DIR-mediated lignin modulation represents a conserved strategy for plants to integrate environmental cues with cell wall remodeling during stress adaptation.

The core function of DIR proteins lies in regulating the biosynthesis of plant secondary metabolites. These proteins participate in the formation of lignin and lignans by mediating radical coupling reactions of monolignols (e.g., coniferyl alcohol). As key components of the secondary cell walls in vascular plants, lignin and lignans collaboratively construct the rigid framework of cell walls, which not only maintains organ morphology and imparts mechanical strength and compression resistance but also participates in biotic and abiotic stress defense [9]. Recent functional studies have demonstrated that DIR genes play multidimensional roles in plant stress resistance mechanisms: for instance, *PIDIR1* and *AtsDIR23* regulate lignin accumulation in *Phryma leptostachya* and *Acorus tatarinowii*, respectively [10,11]. *FvDIR13* enhances disease resistance in strawberry by regulating JA and SA response genes [12]. Overexpression of *GmDIR27* promotes soybean pod dehiscence [13]. *ScDIR7* in sugarcane responds to drought stress [14]. *ZmDIR11* enhances drought tolerance in maize by regulating abscisic acid and lignan metabolism [15]. VvDIR4 in grapevine participates in regulating hormone and lignin biosynthesis pathways for anthracnose resistance [16]. These findings reveal the functional diversity of the DIR family in plant adaptive evolution.

In-depth analysis of the biological functions of the DIR gene family in plant growth and development can not only provide new insights into the molecular regulatory mechanisms of crops responding to abiotic and biotic stresses but also lay an important theoretical foundation for genetic improvement of crop stress resistance. Watermelon (*Citrullus lanatus*) is a representative cucurbit crop of significant economic value. During its growth and development, plants are often challenged by various abiotic and biotic stresses, including high temperature, low temperature, salt, drought, *Fusarium* wilt, powdery mildew, etc. Numerous studies have validated the pivotal role of DIR family genes in plant stress responses. Currently, the functions of DIR genes in responding to abiotic stresses (e.g., drought, salinity) and biotic stresses (e.g., pathogen infection) have been widely studied in various plants such as cassava [17], pigeonpea [18], rice [19], mung bean [20], and *Panax notoginseng* [21]. However, although the genome-wide identification of DIR gene family has been reported in watermelon and their expression patterns under powdery mildew stress were analyzed by quantitative real-time PCR [22], their functions in abiotic stresses and other biotic stresses have not yet been analyzed, and thus their potential roles in these stress responses remain unclear. Therefore, functional characterization of DIR genes in watermelon under diverse stress conditions is crucial for deciphering their specific regulatory roles in stress adaptation mechanisms.

In this study, 22 members of the DIR gene family in watermelon (designated as *ClDIR* genes) were systematically identified through genome-wide screening. Physicochemical properties, chromosomal locations, gene structures, conserved motifs, phylogenetic relationships, *cis*-acting elements, and synteny analysis of *ClDIR* genes were conducted using bioinformatics approaches. Furthermore, based on 21 transcriptome sequencing datasets, tissue-specific expression and stress-responsive expression patterns of *ClDIR* genes were analyzed to preliminarily explore their biological functions in watermelon. Additionally, subcellular localization of the ClDIR8 protein and screening of its interacting proteins were performed. These findings will provide valuable insights for further functional characterization of *ClDIR* genes and offer a theoretical basis for stress-resistant watermelon breeding.

## 2. Results

### 2.1. Genome-Wide Identification of DIR Gene Family in Watermelon

According to the published genomic data of watermelon (97103_v2.5), a total of 22 DIR family members were identified and designated as *ClDIR1*–*ClDIR22*. These genes were unevenly distributed across chromosomes 1, 2, 3, 5, 6, 7, 9, and 10. Chromosome 2 contained the highest number of *ClDIR* genes (8), whereas chromosomes 1 and 3 harbored the fewest (1 *ClDIR* gene each). Coding sequence (CDS) lengths of *ClDIR* genes ranged from 525 bp (*ClDIR3*) to 1200 bp (*ClDIR10*). The deduced ClDIR proteins varied from 174 amino acids (ClDIR3) to 399 amino acids (ClDIR10), with molecular weights spanning 18.44 kDa (ClDIR3) to 43.45 kDa (ClDIR10). Theoretical isoelectric points (pI) ranged from 4.36 (ClDIR6) to 10.04 (ClDIR9). Instability indices of ClDIR proteins varied from 9.42 (ClDIR3) to 54.36 (ClDIR22); proteins with instability indices < 40 were classified as stable, whereas those with instability indices > 40 were considered unstable. Accordingly, *ClDIR6*, *ClDIR10*, *ClDIR13*, *ClDIR14*, *ClDIR17*, and *ClDIR22* encoded unstable proteins, while the remaining *ClDIR* genes encoded stable ClDIR proteins. Aliphatic indices of ClDIR proteins ranged from 68.86 (ClDIR7) to 101.18 (ClDIR9), and grand average of hydropathicity (GRAVY) values spanned −0.337 (ClDIR10) to 0.332 (ClDIR2). Positive GRAVY values indicate hydrophobic tendencies in ClDIR proteins, whereas negative values suggest hydrophilic properties. Thus, *ClDIR4*, *ClDIR6*, *ClDIR7*, *ClDIR10*, *ClDIR13*, and *ClDIR20* encoded hydrophilic ClDIR proteins, while the remaining *ClDIR* genes encoded hydrophobic ClDIR proteins. Notably, most ClDIR proteins had GRAVY values near zero, indicating amphipathic characteristics (Table 1).

### 2.2. Phylogenetic Analysis of ClDIR Genes

**Figure 1:**
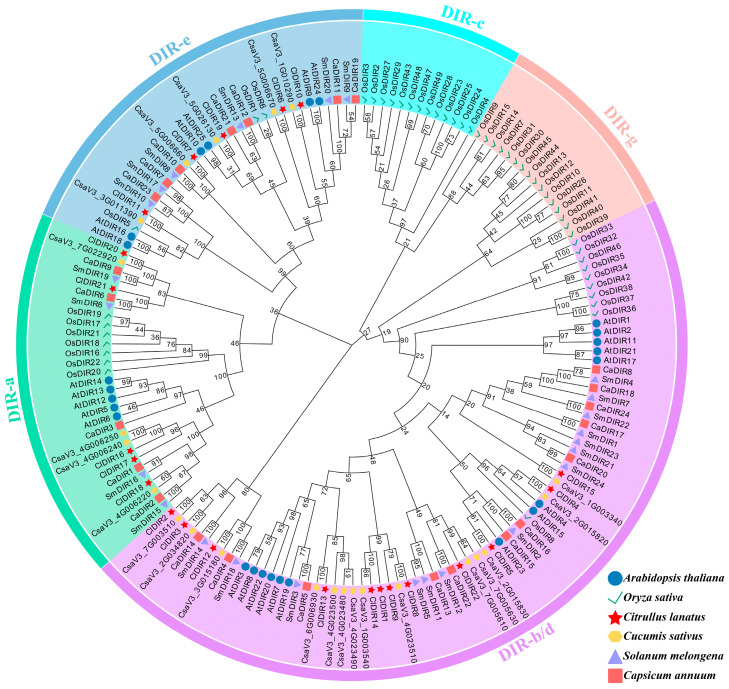
Phylogenetic analysis of DIR proteins from *Arabidopsis*, rice, watermelon, cucumber, eggplant, and pepper.

To decipher the evolutionary trajectories of *ClDIR* genes, a phylogenetic analysis was performed using 167 DIR proteins, including 25 AtDIR proteins from *Arabidopsis*, 49 OsDIR proteins from rice, 24 CaDIR proteins from pepper, 23 CsDIR proteins from cucumber, 24 SmDIR proteins from eggplant, and 22 ClDIR proteins from watermelon. The resultant phylogenetic tree resolved five distinct subgroups: DIR-a, DIR-b/d, DIR-c, DIR-g, and DIR-e. The majority of DIR genes clustered into three subgroups: DIR-a, DIR-b/d, and DIR-e. Notably, the DIR-b/d subgroup contained the highest number of genes (76), suggesting substantial expansion during DIR gene evolution compared to other subgroups. *ClDIR* genes from watermelon were primarily distributed across DIR-a, DIR-b/d, and DIR-e subgroups. Intriguingly, the DIR-c and DIR-g subgroups harbored only *OsDIR* genes from rice, with no representation from other species (Figure 1). These differential distributions imply functional divergence among subgroups. Furthermore, sequence homology analysis revealed strong conservation between *ClDIR* genes and their cucumber orthologs, underscoring the evolutionary preservation of DIR genes across Cucurbitaceae species.

### 2.3. Gene Structure and Conserved Motif Analyses of ClDIR Genes

**Figure 2:**
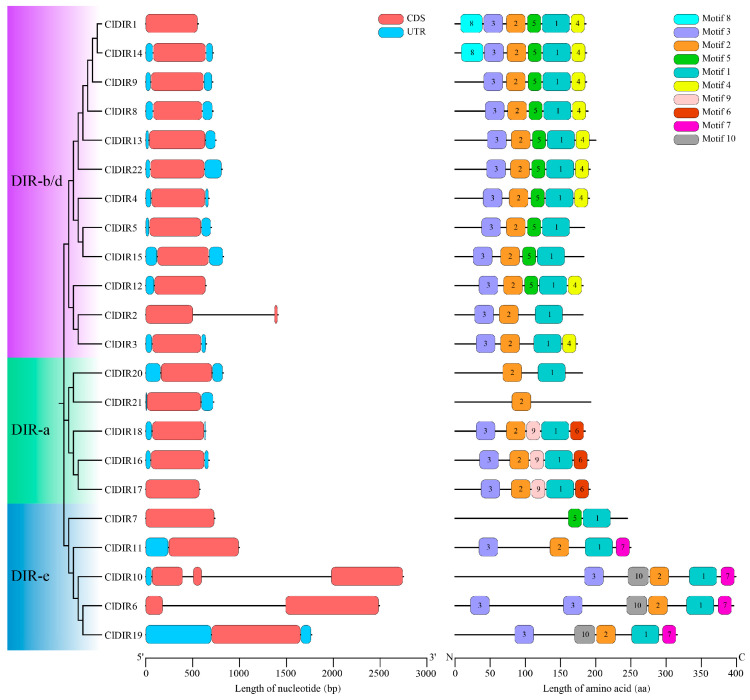
Exon–intron structures of *ClDIR* genes and conserved motifs of ClDIR proteins.

Structural analysis of the 22 *ClDIR* genes revealed three canonical subfamilies (DIR-a, DIR-b/d, and DIR-e) based on phylogenetic clustering, consistent with comparative analyses of watermelon, *Arabidopsis*, rice, eggplant, pepper, and cucumber. Specifically, the DIR-a subfamily comprised 5 *ClDIR* members, DIR-b/d emerged as the largest clade with 12 *ClDIR* genes, and DIR-e contained 5 *ClDIR* genes. Exon–intron architecture analysis showed that most *ClDIR* genes exhibited a typical single-exon structure without introns, though notable variations existed: *ClDIR2* and *ClDIR6* displayed a two-exon structure, while *ClDIR10* harbored a three-exon configuration (Figure 2), potentially reflecting annotation specificity or evolutionary structural remodeling.

Conserved motif analysis of the 22 ClDIR proteins identified 10 motifs with lengths ranging from 21 to 41 amino acids, sequentially designated motif1 to motif10 (Table S1). Subfamily-specific motif distributions were evident: DIR-a members predominantly contained motif1, motif2, motif3, motif6, and motif9; DIR-b/d members typically harbored motif1, motif2, motif3, motif4, and motif5; and DIR-e members were characterized by motif1, motif2, motif3, motif7, and motif10. Notably, motif1 and motif2 were conserved in all ClDIR proteins except ClDIR21 (lacking motif1) and ClDIR7 (lacking motif2) (Figure 2). Pfam annotation revealed that both motif1 and motif2 correspond to the dirigent protein domain, indicating their critical functional significance in the DIR gene family.

### 2.4. Synteny Analysis of ClDIR Genes

**Figure 3:**
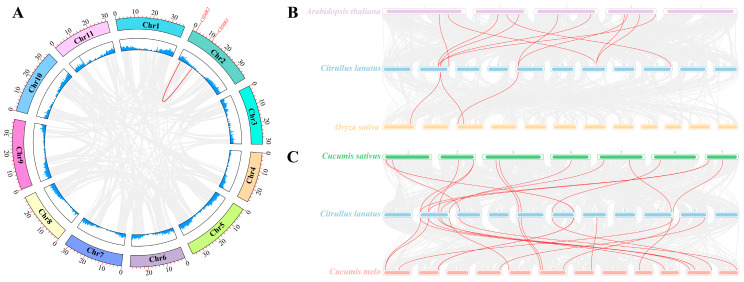
Syntenic relationships of DIR family genes among *Citrullus lanatus* and four representative species. (**A**) Intraspecific collinearity analysis of *ClDIR* genes in *C. lanatus*. (**B**) Interspecific syntenic relationships of DIR genes between *C. lanatus* and two model species (*Arabidopsis thaliana*, *Oryza sativa*). (**C**) Interspecific syntenic relationships of DIR genes between *C. lanatus* and two Cucurbitaceae species (*Cucumis sativus*, *Cucumis melo*).

Collinearity analysis of the *ClDIR* gene family identified four pairs of tandem duplicated genes (*ClDIR4*/*ClDIR5*, *ClDIR6*/*ClDIR7*, *ClDIR8*/*ClDIR9*, *ClDIR16*/*ClDIR17*), accounting for 36.36% of family members, and one pair of segmental duplicated genes (*ClDIR2*/*ClDIR3*) (Figure 3A), comprising 9.09% of the family. These results indicated that tandem duplication was the primary driver of rapid expansion and evolution in the watermelon DIR gene family. Ka/Ks (non-synonymous substitution rates/synonymous substitution rates) ratio analysis of duplicated gene pairs revealed that three tandem duplicated pairs (*ClDIR4*/*ClDIR5*, *ClDIR8*/*ClDIR9*, *ClDIR16*/*ClDIR17*) and one segmental pair (*ClDIR2*/*ClDIR3*) had Ka/Ks < 1, suggesting purifying selection and conserved functions. By contrast, the tandem pair ClDIR6/ClDIR7 exhibited Ka/Ks > 1, indicating positive selection (Table 2).

To trace evolutionary trajectories, interspecific collinearity analysis was conducted between *ClDIR* genes in watermelon and DIR genes from the dicotyledonous plant *Arabidopsis thaliana* and the monocotyledonous plant rice (*Oryza sativa*). Genomic comparisons revealed 3 DIR orthologs between watermelon and rice and 12 between watermelon and *Arabidopsis* (Figure 3B), indicating a closer divergence time between watermelon and *Arabidopsis* relative to rice. Collinearity analyses with other cucurbit species showed 15 collinear gene pairs between watermelon and cucumber and 17 between watermelon and melon (Figure 3C). Notably, *ClDIR6* and *ClDIR11* exhibited collinearity with all analyzed species. Additionally, *ClDIR8* and *ClDIR19* also showed collinearity with *Arabidopsis*, cucumber, and melon (Table S2). These findings suggest that *ClDIR6*, *ClDIR8*, *ClDIR11*, and *ClDIR19* may represent ancestral genes conserved across watermelon, cucumber, and melon, retaining similar structures and functions through shared chromosomal rearrangements. Variations in collinear pair numbers among species likely stem from evolutionary gene rearrangement, deletion, or insertion events.

### 2.5. Analysis of Cis-Acting Elements in ClDIR Promoters

**Figure 4:**
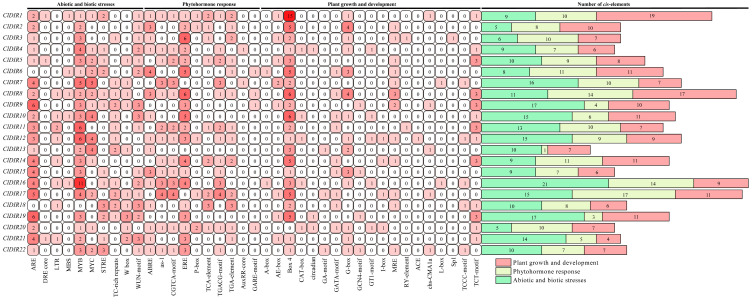
*Cis*-elements analysis of the promoters of *ClDIR* genes. Different shades of red indicate the number of *cis*-acting elements, with deeper colors corresponding to higher counts.

*Cis*-acting element analysis of *ClDIR* gene promoters identified 39 distinct types of regulatory motifs, systematically categorized into three functional classes: abiotic and biotic stress-responsive *cis*-acting elements, phytohormone response-related *cis*-acting elements, and plant growth and development-related *cis*-acting elements (Figure 4).

Among the abiotic and biotic stress-responsive *cis*-acting elements, 10 key motifs (ARE, DRE core, LTR, MBS, MYB, MYC, STRE, TC-rich repeats, W box, and WUN-motif) were identified, mediating responses to oxidative, drought, low-temperature, osmotic, high-temperature stresses, and wound signaling. The MYB transcription factor binding site exhibited the highest prevalence, detected in 21 *ClDIR* promoters, followed by the ARE element (anaerobic response under waterlogging), present in 20 *ClDIR* promoters.

In the phytohormone response-related *cis*-acting elements, 10 characteristic motifs (ABRE, as-1, CGTCA-motif, ERE, P-box, TCA-element, TGACG-motif, TGA-element, AuxRR-core, and GARE-motif) regulated signaling pathways for abscisic acid (ABA), salicylic acid (SA), methyl jasmonate (MeJA), ethylene, gibberellin (GA), and auxin. The ethylene-responsive element (ERE) was most widely distributed, occurring in 86% of *ClDIR* promoters.

In the plant growth and development-related *cis*-acting elements, 19 motifs (A-box, AE-box, Box 4, CAT-box, circadian, GA-motif, GATA-motif, G-box, GCN4-motif, GT1-motif, I-box, MRE, RY-element, ACE, chs-CMA1a, L-box, Sp1, TCCC-motif, TCT-motif) governed light signal transduction, cell cycle regulation, seed germination, stem elongation, and circadian rhythms. All *ClDIR* promoters contained light-responsive elements, with Box 4 (95%) the most prevalent, followed by G-box (64%) and TCT-motif (59%).

Collectively, 646 *cis*-acting elements were identified across *ClDIR* promoters. *ClDIR8*, *ClDIR16*, and *ClDIR17* harbored the richest element diversity (42, 44, and 43 elements, respectively), whereas *ClDIR13* had only 18 elements, highlighting substantial variation in *cis*-element composition. This variability suggested diverse transcriptional regulation and functional specialization among *ClDIR* genes.

### 2.6. Tissue-Specific Expression Analysis of ClDIR Genes

**Figure 5:**
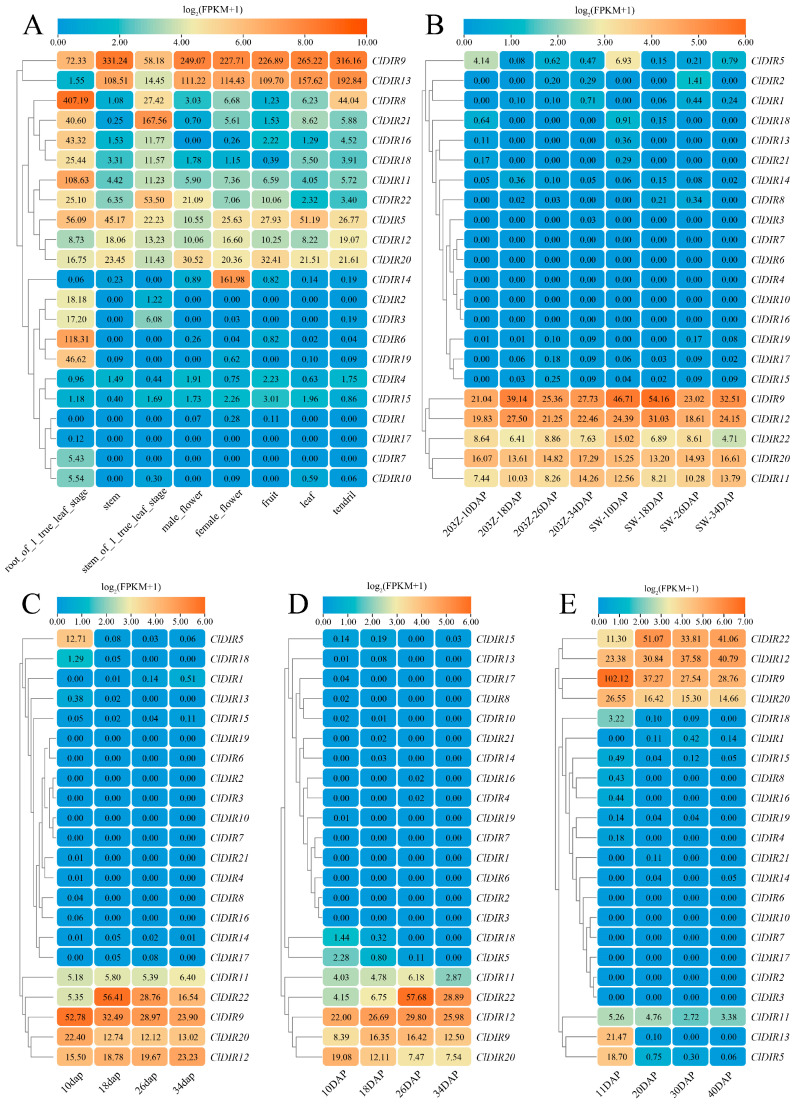
Expression heatmap of *ClDIR* genes in diverse tissues (**A**) and fruit developmental stages (**B**–**E**) of watermelon. 203Z: sweet watermelon; SW: sour watermelon; DAP or dap: days after pollination. The data in the boxes indicate the original FPKM values.

Tissue-specific expression analysis revealed that the *ClDIR9* gene exhibited high expression in watermelon roots, stems, flowers, fruits, leaves, and tendrils. A subset of *ClDIR* genes (*ClDIR4*, *ClDIR15*, *ClDIR1*, *ClDIR17*, *ClDIR7*, and *ClDIR10*) displayed no expression or extremely low expression levels across all detected tissues. The *ClDIR13* gene was expressed in all tissues but showed reduced expression in true leaf-stage roots. *ClDIR8* and *ClDIR11* were universally expressed with specific high expression in true leaf-stage roots. The *ClDIR21* gene was expressed in all tissues, peaking in true leaf-stage stems. Additionally, *ClDIR16* was expressed in all tissues except male flowers, with the highest expression in true leaf-stage roots. *ClDIR18* showed universal expression, peaking in true leaf-stage roots and being the lowest in fruits. *ClDIR22* was expressed in all tissues, highest in true leaf-stage stems and lowest in leaves. *ClDIR5* showed universal expression, highest in true leaf-stage roots and lowest in male flowers. *ClDIR12* was expressed in all tissues, highest in tendrils and lowest in leaves. *ClDIR20* showed universal expression, highest in fruits and lowest in true leaf-stage stems. Notably, *ClDIR14* exhibited specific expression in female flowers, while *ClDIR2*, *ClDIR3*, *ClDIR6*, and *ClDIR19* showed tissue-specific expression in true leaf-stage roots (Figure 5A).

During fruit development, *ClDIR9*, *ClDIR11*, *ClDIR12*, *ClDIR20*, and *ClDIR22* maintained high expression at 10, 18, 26, and 34 days after pollination (DAP). In contrast, 15 genes (*ClDIR1*, *ClDIR2*, *ClDIR3*, *ClDIR4*, *ClDIR6*, *ClDIR7*, *ClDIR8*, *ClDIR10*, *ClDIR14*, *ClDIR15*, *ClDIR16*, *ClDIR17*, *ClDIR18*, *ClDIR19*, and *ClDIR21*) were either non-expressed or showed minimal expression during these stages. The *ClDIR5* gene displayed specific expression at 10 DAP (Figure 5B–E). Collectively, these results demonstrated that *ClDIR* genes exhibited distinct expression patterns across tissues and developmental stages, highlighting their tissue-specific and spatiotemporal expression characteristics.

### 2.7. Expression Analysis of ClDIR Genes Under Abiotic Stresses

**Figure 6:**
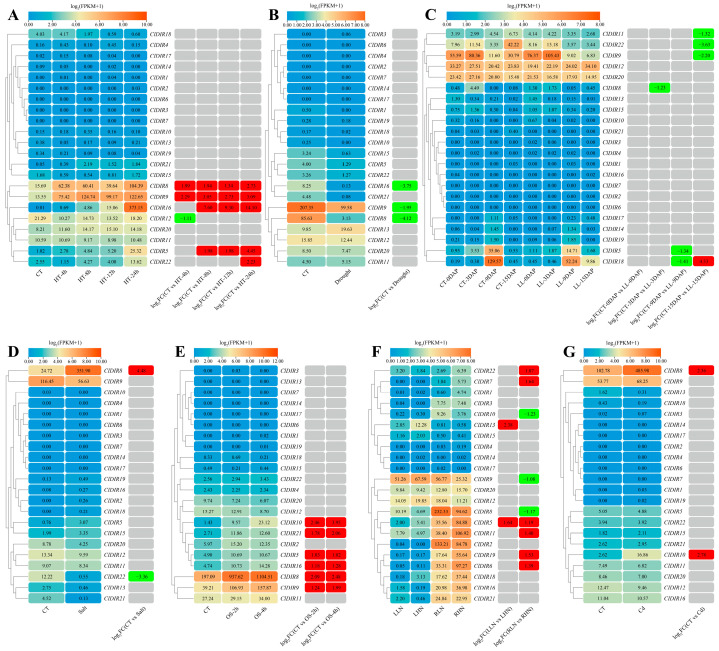
The expression heatmaps of *ClDIR* genes under abiotic stresses in watermelon. (**A**) The expression patterns of *ClDIR* genes under high-temperature stress. CT: Control treatment; HT-4h, HT-8h, HT-12h, and HT-24h: high-temperature treatment for 4, 8, 12, and 24 h. (**B**) The expression patterns of *ClDIR* genes under drought stress. (**C**) The expression patterns of *ClDIR* genes under low-light stress. CT: Control treatment; LL: low light; 0DAP, 3DAP, 9DAP, and 15DAP: 0, 3, 9, and 15 days after pollination. (**D**) The expression patterns of *ClDIR* genes under salt stress. (**E**) The expression patterns of *ClDIR* genes under osmotic stress. CT: Control treatment; OS-2h and OS-4h: osmotic stress for 2 and 4 h. (**F**) The expression patterns of *ClDIR* genes under nitrogen treatment. LLN: leaf low nitrogen; LHN: leaf high nitrogen; RLN: root low nitrogen; RHN: root high nitrogen. (**G**) The expression patterns of *ClDIR* genes under cadmium stress. CT: Control treatment; Cd: cadmium stress. Original FPKM values were shown in heatmap boxes; differentially expressed genes were highlighted in red (upregulation) and green (downregulation) with log_2_(fold-change) values.

Expression pattern analysis based on transcriptome sequencing data showed that under high-temperature stress, only the *ClDIR12* gene was significantly downregulated, while *ClDIR8*, *ClDIR9*, *ClDIR16*, *ClDIR5*, and *ClDIR22* were significantly upregulated. *ClDIR8* and *ClDIR9* were upregulated at 4, 8, 12, and 24 h post-stress. *ClDIR16* and *ClDIR5* were significantly upregulated at 8, 12, and 24 h. *ClDIR22* was only upregulated at 24 h, while the remaining 16 *ClDIR* genes showed no significant expression changes (Figure 6A). Under drought stress, *ClDIR16*, *ClDIR9*, and *ClDIR8* were significantly downregulated, and the other 19 *ClDIR* genes showed no changes (Figure 6B). Under low-light treatment, *ClDIR18* was significantly downregulated at 9 DAP and upregulated at 15 DAP. *ClDIR11*, *ClDIR22*, *ClDIR9*, *ClDIR8*, and *ClDIR5* were significantly downregulated. *ClDIR11*, *ClDIR22*, and *ClDIR9* were downregulated only at 15 DAP, and *ClDIR8* and *ClDIR5* were downregulated only at 9 DAP. The remaining 16 *ClDIR* genes were unaffected (Figure 6C). Under salt stress, *ClDIR8* was significantly upregulated, *ClDIR22* was downregulated, and the other 20 *ClDIR* genes showed no expression differences (Figure 6D). During osmotic stress, *ClDIR10*, *ClDIR21*, *ClDIR5*, *ClDIR16*, *ClDIR8*, and *ClDIR9* were significantly upregulated at 2 and 4 h post-stress, while the remaining 16 *ClDIR* genes showed no response (Figure 6E). Under high-nitrogen stress, in roots, *ClDIR22*, *ClDIR7*, *ClDIR11*, *ClDIR19*, and *ClDIR6* were significantly upregulated, whereas *ClDIR10*, *ClDIR9*, and *ClDIR8* were downregulated. *ClDIR13* was upregulated in leaves, and *ClDIR5* was upregulated in both leaves and roots. The other 12 *ClDIR* genes were unchanged (Figure 6F). Under cadmium stress, *ClDIR8* and *ClDIR10* were significantly upregulated, and the remaining 20 *ClDIR* genes showed no response (Figure 6G).

### 2.8. Expression Analysis of ClDIR Genes Under Biotic Stresses

**Figure 7:**
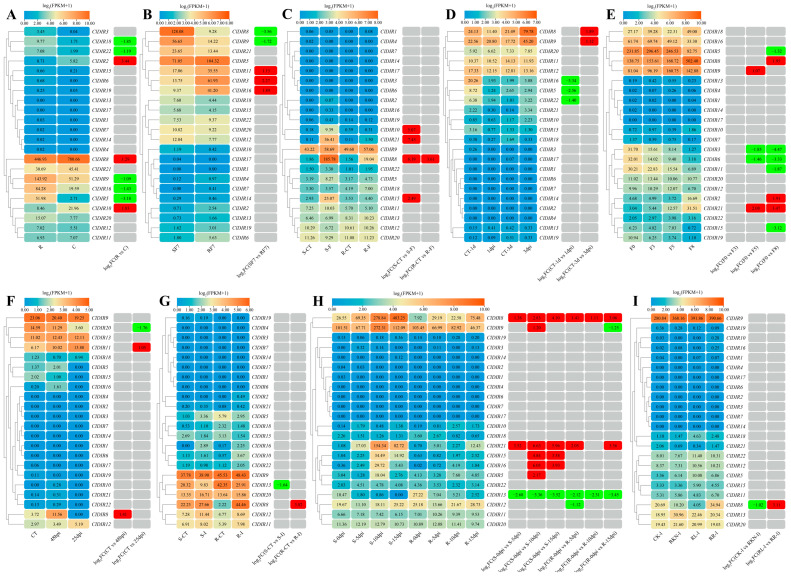
The expression heatmaps of *ClDIR* genes under biotic stresses in watermelon. (**A**–**E**) The expression patterns of *ClDIR* genes under *Fusarium* wilt stress. R: oilseed rape rotation cropping; C: continuous watermelon monocropping; SF7: susceptible cultivar at 7 days post inoculation with *Fusarium oxysporum* f. sp. *niveum*; RF7: resistant cultivar at 7 days post inoculation with *Fusarium oxysporum* f. sp. *Niveum*; S-CT: susceptible cultivar under non-inoculated control conditions; S-F: susceptible cultivar inoculated with *Fusarium oxysporum* f. sp. *niveum*; R-CT: resistant cultivar under non-inoculated control conditions; S-F: resistant cultivar inoculated with *Fusarium oxysporum* f. sp. *niveum*; CT-1d: non-inoculated control plants at 1 day; 1 dpi: plants at 1 day post inoculation with *Fusarium oxysporum* f. sp. *niveum*; CT-3d: non-inoculated control plants at 3 days; 3 dpi: plants at 3 days post inoculation with *Fusarium oxysporum* f. sp. *niveum*; F0, F3, F5, and F8: 0, 3, 5, and 8 days after inoculation with *Fusarium oxysporum* f. sp. *niveum*. (**F**) The expression patterns of *ClDIR* genes under cucumber green mottle mosaic virus stress. CT: control treatment; 48 hpi and 25 dpi: 48 h and 25 days post inoculation. (**G**) The expression patterns of *ClDIR* genes under powdery mildew stress. S-CT: non-inoculated control of susceptible cultivar; S-I: susceptible cultivar inoculated with powdery mildew; R-CT: non-inoculated control of resistant cultivar; R-I: resistant cultivar inoculated with powdery mildew. (**H**) The expression patterns of *ClDIR* genes under squash vein yellowing virus stress. S: susceptible plants; R: resistant plants; 0 dpi, 5 dpi, 10 dpi, and 15 dpi were 0, 5, 10, and 15 days post inoculation, respectively. (**I**) The expression patterns of *ClDIR* genes under root-knot nematode stress. CK-l: leaf samples under white light with water treatment; RKN-l: leaf samples under white light inoculated with *Meloidogyne incognita*; RL-l: leaf samples under red light with water treatment; RR-l: leaf samples under red light inoculated with *Meloidogyne incognita*. Original FPKM values are shown in heatmap boxes; differentially expressed genes are highlighted in red (upregulation) and green (downregulation) with log_2_(fold-change) values.

Transcriptome sequencing data of five sets of watermelons under *Fusarium* wilt stress were analyzed to explore the expression patterns of *ClDIR* genes. The results showed that compared with watermelon–oilseed rape rotation (R), the expressions of *ClDIR2*, *ClDIR8*, and *ClDIR10* were significantly upregulated, while the expressions of *ClDIR5*, *ClDIR9*, *ClDIR16*, *ClDIR18*, and *ClDIR22* were significantly downregulated under continuous watermelon monocropping (C) (Figure 7A). When compared with the susceptible materials 7 days after *Fusarium* wilt inoculation (SF7), the expression levels of *ClDIR11*, *ClDIR3*, and *ClDIR16* were significantly upregulated, and the expression levels of *ClDIR8* and *ClDIR9* were significantly downregulated in the resistant materials 7 days after *Fusarium* wilt inoculation (RF7) (Figure 7B). After inoculation with *Fusarium* wilt, the susceptible materials (S-F) and resistant materials (R-F) were compared with their respective controls (S-CT and R-CT). The expression levels of *ClDIR10*, *ClDIR21*, and *ClDIR15* were only significantly upregulated in the susceptible materials, while the expression level of *ClDIR8* was significantly upregulated in both resistant and susceptible materials (Figure 7C). Time-course analysis showed that *ClDIR5*, *ClDIR16*, and *ClDIR22* were downregulated at 1 day post inoculation (1 dpi), and *ClDIR8* and *ClDIR9* were repressed at 3 dpi relative to controls (CT-1d, CT-3d) (Figure 7D). At 8 days post inoculation with *Fusarium* wilt (F8), *ClDIR5*, *ClDIR11*, and *ClDIR15* were specifically downregulated compared with the control (F0), while *ClDIR8* and *ClDIR2* were induced. *ClDIR9* was upregulated only at 5 days post inoculation (F5). *ClDIR3* and *ClDIR6* were repressed at both F5 and F8, whereas *ClDIR21* was consistently upregulated at both time points (Figure 7E).

Transcriptome sequencing data of watermelons under stresses of cucumber green mottle mosaic virus, powdery mildew, squash vein yellowing virus, and root-knot nematodes were further utilized to analyze the expression patterns of *ClDIR* genes under biotic stresses. Under cucumber green mottle mosaic virus stress, *ClDIR8* was significantly upregulated at 48 dpi, while *ClDIR11* was induced at 25 dpi and *ClDIR20* was repressed at the same time point (Figure 7F). For powdery mildew stress, inoculation of susceptible and resistant materials revealed that *ClDIR13* was downregulated in susceptible lines, whereas *ClDIR8* was upregulated in resistant genotypes relative to controls (Figure 7G). Under squash vein yellowing virus stress, *ClDIR8* and *ClDIR21* were upregulated in both resistant and susceptible materials, while *ClDIR13* was downregulated in both resistant and susceptible lines. *ClDIR15*, *ClDIR16*, and *ClDIR5* were uniquely upregulated in susceptible lines, and *ClDIR12* was specifically downregulated in resistant lines. Notably, *ClDIR9* was induced in susceptible materials but repressed in resistant materials (Figure 7H). For root-knot nematode stress, *Meloidogyne incognita* infection decreased *ClDIR8* expression in watermelon leaves under white light compared with water-treated controls, but induced *ClDIR8* under red-light treatment relative to red-light-treated controls (Figure 7I).

### 2.9. Expression Patterns of ClDIR Genes Under Abiotic and Biotic Stresses

**Figure 8:**
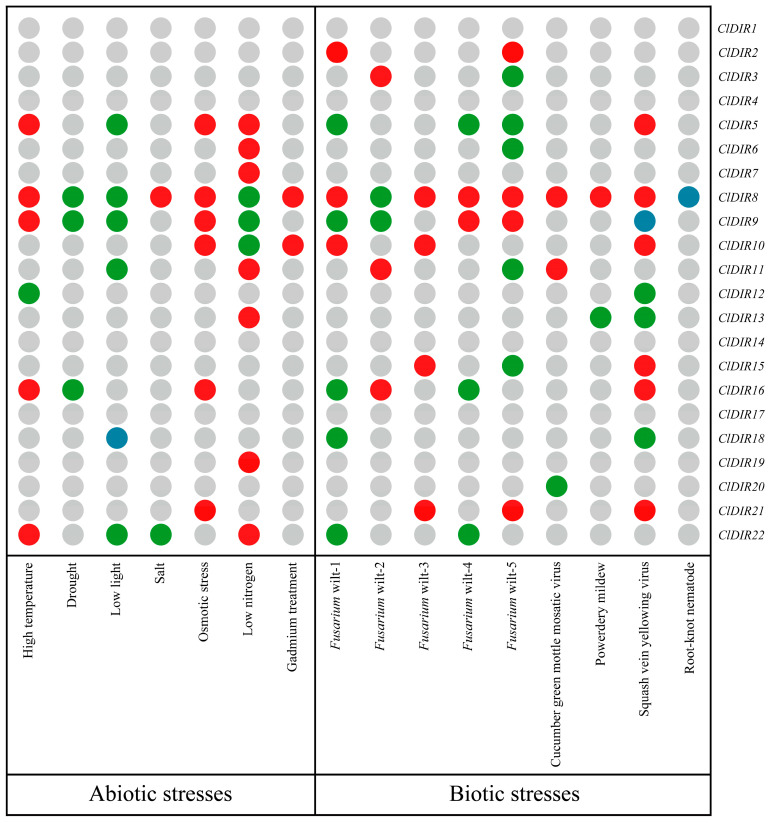
The heatmap for expression patterns of *ClDIR* genes under abiotic and biotic stresses. Gray indicates no differential expression; red indicates upregulation; green indicates downregulation; and blue indicates both up- and downregulation.

To analyze the expression patterns of *ClDIR* genes under abiotic and biotic stresses, differentially expressed genes were marked and visualized as a heatmap (Figure 8). Results showed that 18 of the 22 *ClDIR* genes exhibited stress responses, with only 4 genes (*ClDIR1*, *ClDIR4*, *ClDIR14*, *ClDIR17*) showing no response. Notably, *ClDIR8* displayed significant differential expression across all 16 abiotic and biotic stress conditions. *ClDIR5* responded significantly to four abiotic and four biotic stresses. *ClDIR9* was differentially expressed in five abiotic and five biotic stresses. Some DIR genes responded exclusively to abiotic stresses, such as *ClDIR6*, *ClDIR7*, and *ClDIR19*, which were upregulated only under nitrogen treatment. In contrast, genes like *ClDIR2*, *ClDIR3*, and *ClDIR15* showed differential expression only under biotic stresses, while *ClDIR20* was downregulated exclusively under cucumber green mottle mosaic virus stress. The remaining 11 *ClDIR* genes responded to both stress types. Significantly, *ClDIR8* was expressed in all 16 stress conditions, indicating its critical research value in regulating biotic and abiotic stress responses.

### 2.10. Validation of the Expression Profiles of ClDIR Genes Under High-Temperature, Drought, Salt and Low-Temperature Stresses

To elucidate the functional roles of *ClDIR* genes in responding to abiotic stresses, six candidate *ClDIR* genes (*ClDIR5*, *ClDIR8*, *ClDIR9*, *ClDIR12*, *ClDIR16*, *ClDIR22*) with significant differential expression under abiotic stresses were selected based on transcriptome analysis. qRT-PCR was then employed to analyze their temporal dynamic expression patterns (0, 6, 12, and 24 h) in roots and leaves in response to four abiotic stresses: high-temperature (45 °C), drought (20% PEG6000), salt (500 mmol·L^−1^ NaCl), and low-temperature (4 °C).

**Figure 9:**
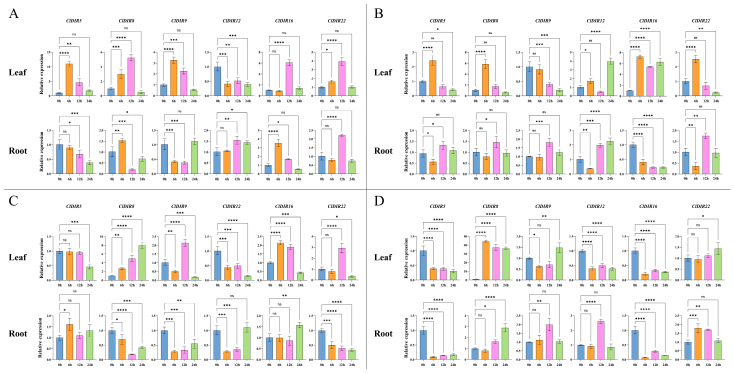
Expression profiles of *ClDIR* genes in response to diverse abiotic stresses. (**A**) Expression profiles of *ClDIR* genes under high-temperature stress (45 °C). (**B**) Expression profiles of *ClDIR* genes under drought stress (20% PEG6000). (**C**) Expression profiles of *ClDIR* genes under salt stress (500 mmol·L^−1^ NaCl). (**D**) Expression profiles of *ClDIR* genes under low-temperature stress (4 °C). Data are presented as means ± SE (*n* = 3) from three independent biological replicates. Statistical significance is denoted as ns (*p* > 0.05, not significant), * (*p* < 0.05), ** (*p* < 0.01), *** (*p* < 0.001), and **** (*p* < 0.0001).

Under high-temperature stress, the expressions of *ClDIR* genes exhibited tissue-specific divergence. In leaves, *ClDIR5*, *ClDIR8*, *ClDIR9*, and *ClDIR22* displayed transient upregulation at 6–12 h, reverting to the 0 h levels by 24 h; *ClDIR12* showed persistent downregulation from 6 to 24 h; and *ClDIR16* was only upregulated at 12 h. In roots, *ClDIR5* was downregulated at 12–24 h; *ClDIR8* presented early upregulation at 6 h followed by downregulation; *ClDIR9* decreased at 6–12 h and then recovered; *ClDIR12* remained static at 6 h but was upregulated at 12–24 h; *ClDIR16* showed upregulation at 6 h and downregulation at 12 h; and *ClDIR22* was only upregulated at 12 h (Figure 9A).

Under drought stress, tissue-specific expression patterns emerged. In leaves, *ClDIR5*, *ClDIR8*, *ClDIR12*, and *ClDIR22* showed stage-specific upregulation (at 6 h or 24 h); *ClDIR9* was downregulated at 12–24 h; and *ClDIR16* was consistently upregulated from 6 to 24 h. In roots, *ClDIR5* was downregulated at 6 h and upregulated at 12 h; *ClDIR8* and *ClDIR9* were only upregulated at 12 h; *ClDIR12* was downregulated at 6 h and upregulated at 12–24 h; *ClDIR16* was consistently downregulated from 6 to 24 h; and *ClDIR22* was downregulated at 6 h, upregulated at 12 h, and recovered to the 0 h level at 24 h (Figure 9B).

Under salt stress, distinct tissue-specific responses were observed. In leaves, *ClDIR5* was downregulated at 24 h; *ClDIR8* was consistently upregulated from 6 to 24 h; and *ClDIR9*, *ClDIR12*, *ClDIR16*, and *ClDIR22* exhibited bidirectional expression changes (e.g., *ClDIR9* was downregulated at 6 h, upregulated at 12 h, and downregulated again at 24 h). In roots, *ClDIR5* was upregulated at 6 h; *ClDIR8*, *ClDIR9*, and *ClDIR22* were consistently downregulated; *ClDIR12* was downregulated at 6–12 h and recovered to the 0 h level at 24 h; and *ClDIR16* was upregulated only at 24 h (Figure 9C).

Under low-temperature stress, tissue-specific divergence of *ClDIR* expression occurred. In leaves, *ClDIR5*, *ClDIR12*, and *ClDIR16* were consistently downregulated from 6 to 24 h; *ClDIR8* was consistently upregulated; *ClDIR9* was downregulated at 6 h, showed recovery at 12 h, and was upregulated at 24 h; and *ClDIR22* was upregulated only at 24 h. In roots, *ClDIR5* and *ClDIR16* were consistently downregulated; *ClDIR9* and *ClDIR12* were upregulated only at 12 h; *ClDIR8* was upregulated at 12–24 h; and *ClDIR22* was upregulated at 6–12 h and recovered to the 0 h level at 24 h (Figure 9D).

### 2.11. Subcellular Localization of ClDIR8 Protein

**Figure 10:**
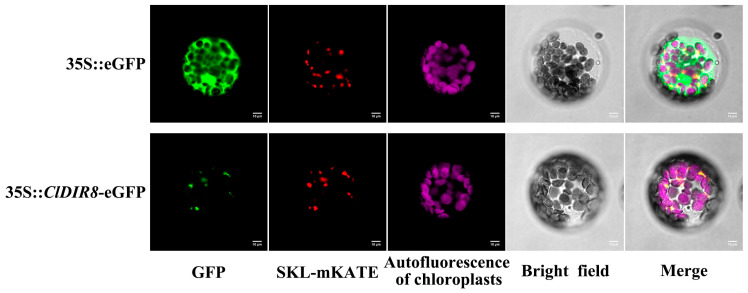
Subcellular localization analysis of ClDIR8 protein.

To characterize the subcellular localization of ClDIR8 protein, the 35S::*ClDIR8*-eGFP fusion expression vector was constructed. The PEG-mediated transient transformation method was employed to introduce the 35S::*ClDIR8*-eGFP recombinant plasmid, the control plasmid 35S::eGFP, and the peroxisomal marker plasmid 35S::SKL-mKate into *Arabidopsis* protoplasts. Fluorescence imaging analysis revealed that the 35S::*ClDIR8*-eGFP fusion protein was specifically localized to peroxisomes (Figure 10). This result indicated that ClDIR8 protein was a peroxisome-localized protein, providing important clues for elucidating its biological function.

### 2.12. Screening of Proteins Interacting with ClDIR8 Protein

**Figure 11:**
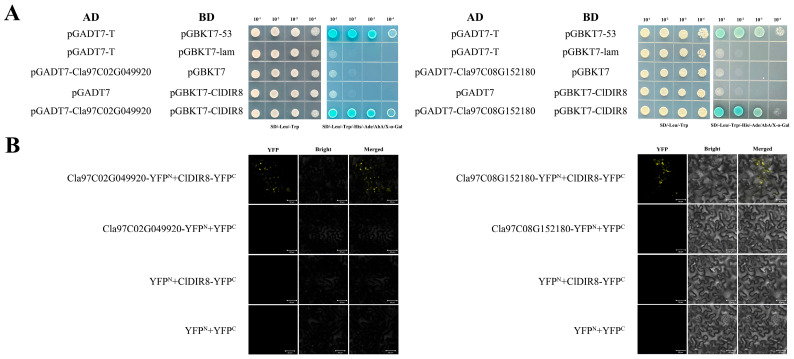
Validation of ClDIR8 protein interactions via Y2H and BiFC assays. (**A**) Y2H confirmation of physical interactions between ClDIR8 and candidate proteins. (**B**) BiFC visualization of ClDIR8 interactions in *Nicotiana benthamiana* leaves.

To dissect the molecular interaction network of the *ClDIR8* gene, a yeast two-hybrid (Y2H) library was constructed for initial screening of interacting proteins, yielding 81 positive clones (Figure S1). Following Sanger sequencing and BLAST homology analysis, 50 candidate interacting proteins were identified (Table S3). For verification, *ClDIR8* and candidate genes were cloned into pGBKT7 and pGADT7 vectors, respectively. Co-transformed yeast strains were assayed on SD-Leu-Trp and SD-Leu-Trp-His-Ade + X-α-Gal plates. Combinations of pGBKT7-*ClDIR8* + pGADT7-*Cla97C02G049920* and pGBKT7-*ClDIR8* + pGADT7-*Cla97C08G152180* grew and turned blue on SD-Leu-Trp-His-Ade + X-α-Gal plates (Figure 11A), indicating transcriptional activation interactions between ClDIR8 protein and these two candidates.

Subsequently, YFP fusion vectors of *ClDIR8* and candidates were constructed in pCAMBIA1300-35S-N and pCAMBIA1300-35S-C, respectively, and co-transformed into *Nicotiana benthamiana* leaves via *Agrobacterium tumefaciens* GV3101-mediated infiltration. Laser confocal microscopy revealed YFP (yellow fluorescent protein) fluorescence complementation signals for both *ClDIR8*/*Cla97C02G049920* and *ClDIR8*/*Cla97C08G152180* combinations, whereas negative controls (empty vectors and non-fused YFP^N^/YFP^C^) showed no obvious fluorescence (Figure 11B), verifying interactions at the subcellular level. In conclusion, via Y2H and bimolecular fluorescence complementation (BiFC) cross-verification, *Cla97C02G049920* (encoding peroxidase) and *Cla97C08G152180* (encoding catalase) were confirmed as genuine ClDIR8-interacting proteins.

## 3. Discussion

### 3.1. Identification of DIR Gene Family in Watermelon and Its Evolutionary Context

The dirigent protein was initially discovered to regulate the regional and stereoselective coupling reaction of lignin biosynthesis [23]. Recent studies have shown that DIR proteins can control the formation of specific chemical bonds during the polymerization of monolignols into lignin polymers. These DIR proteins not only promote plant lignification but also enable plants to respond to biotic and abiotic stresses, playing a central role in biotic resistance and abiotic adaptation [24]. Currently, the DIR gene family has been identified in a variety of plant species. In monocotyledons, it includes 49 *DIR* members in *Oryza sativa* [19], 38 in *Panicum italicum* [25], and 47 in *Phyllostachys edulis* [26]. In dicotyledons, there are 25 in *Arabidopsis thaliana* [27], 24 in *Capsicum annuum* [28], 24 in *Solanum melongena* [29], 25 in *Cajanus cajan* [18], and 23 in *Cucumis sativus* [30]. In this study, 22 *ClDIR* family genes were successfully identified from the watermelon genome using bioinformatics approaches, and the number of identified *ClDIR* genes is consistent with previous research findings. Compared with the above-identified species, the number of *ClDIR* genes was significantly lower than that in monocots but similar to that in dicot crops. This difference suggests that the DIR gene family may have undergone lineage-specific adaptive expansion during the evolution of monocots.

### 3.2. Mechanisms of DIR Gene Family Expansion in Watermelon

The expansion of gene families mainly relies on three molecular mechanisms: tandem duplication, segmental duplication, and transposition [31]. For example, in *Medicago truncatula*, 45 *MtDIR* genes have been identified, and 82.22% (37) of *MtDIR* genes arose from tandem duplication events [32]. In *Phyllostachys edulis*, 47 *PeDIR* genes have been identified, with 67.6% (32) of *PeDIR* genes resulting from tandem duplications and 10 pairs of segmental duplications identified [26]. These results indicate that numerous duplication events within species drive the expansion of DIR family members. In this study, four pairs of tandemly duplicated genes (*ClDIR4*/*ClDIR5*, *ClDIR6*/*ClDIR7*, *ClDIR8*/*ClDIR9*, *ClDIR16*/*ClDIR17*) were found in the watermelon DIR gene family, comprising 36.36% of the gene family, and one pair of segmentally duplicated genes (*ClDIR2*/*ClDIR3*), accounting for 9.09%. Therefore, tandem duplication likely served as the primary driving force for the expansion of *ClDIR* genes.

### 3.3. Phylogenetic Classification and Subfamily-Specific Features of ClDIR Genes

A phylogenetic tree constructed from 167 DIR proteins across *Arabidopsis thaliana*, *Oryza sativa*, *Citrullus lanatus* (watermelon), *Cucumis sativus*, *Solanum melongena*, and *Capsicum annuum* classified DIR proteins into five subfamilies: DIR-a, DIR-b/d, DIR-c, DIR-e, and DIR-g. Notably, watermelon *ClDIR* genes were exclusively distributed in DIR-a, DIR-b/d, and DIR-e subfamilies, with no members detected in DIR-c or DIR-g. The DIR-a subfamily has a well-established functional research history: its members were first linked to lignin biosynthesis in 1997 [33], and in 2012, biochemical analysis of recombinant AtDIR5 and AtDIR6 proteins showed they synergize with laccase to drive oxidative coupling of coniferyl alcohol into (−)-pinoresinol in vitro [34]. Five watermelon genes (*ClDIR16*, *ClDIR17*, *ClDIR18*, *ClDIR20*, *ClDIR21*) clustered with *AtDIR5*/*AtDIR6* in DIR-a, suggesting their potential role in lignin synthesis. DIR-b/d subfamily proteins mediate both abiotic and biotic stress responses [35,36], and previous studies as well as this clustering analysis confirm DIR-b/d as the largest DIR subfamily, implying broad functional diversity. Conversely, DIR-c is monocot-specific, featuring low sequence conservation and a ~140-amino-acid C-terminal extension similar to Jacalin-like domains [37]. This domain confers defense functions and abiotic stress (salt/drought) responsiveness [38], and may contribute to insect resistance [39] and disease resistance [40,41]. Consistent with prior reports [22,37], DIR-c members were detected only in *O. sativa* among the studied species, absent in all dicots including watermelon.

### 3.4. Gene Structure and Conserved Motifs of ClDIR Genes

The typical structure of DIR genes usually contains only one exon [37]. Analysis of the gene structure of watermelon *ClDIR* genes showed that 86.4% (19) of the *ClDIR* genes retained the canonical single-exon architecture, while only 3 genes (*ClDIR2*, *ClDIR6*, *ClDIR10*) contained introns. Notably, all members of the DIR-a subfamily maintained the single-exon structure, indicating higher structural conservation of DIR-a compared to the other subfamilies described earlier. It has been proposed that introns may delay regulatory responses, whereas intron-lacking genes accelerate transcription rates, enabling plants to promptly respond to stresses [42]. Additionally, reduced intron numbers may enhance gene function, improving plant tolerance to various stresses [43]. Conserved motifs (e.g., motif1, motif2, motif3) were identified in the majority of *ClDIR* genes, reflecting their structural conservation. However, subfamily-specific motifs were also observed: motif9 was exclusive to the DIR-a subfamily, and motif10 was unique to the DIR-e subfamily, highlighting functional diversification across DIR subfamilies.

### 3.5. Cis-Acting Elements in ClDIR Promoters

Bioinformatics analysis of *cis*-acting elements in the 2000 bp promoter region upstream of *ClDIR* genes identified multiple *cis*-acting elements associated with hormone responses (e.g., ABRE, as-1, CGTCA-motif, ERE), abiotic and biotic stresses (e.g., STRE, MBS), and growth and development (e.g., G-box, AE-box). This indicates that the *ClDIR* genes play a significant role in the growth, development, and stress responses of watermelon. Among them, 59.1% (13) of *ClDIR* genes contain *cis*-acting elements responsive to methyl jasmonate (MeJA) regulation (i.e., CGTCA-motif or TGACG-motif), and a high proportion of 68.18% (15) of *ClDIR* genes possess ABRE elements responsive to abscisic acid (ABA) regulation. Relevant studies have shown that hormones such as MeJA and ABA can enhance the activities of plant cell protective enzymes (such as POD, CAT, and SOD), scavenge free radicals, and increase the content of osmotic adjustment substances, thereby reducing stress damage to plants [44]. Therefore, it is speculated that *ClDIR* genes may have functions related to plant hormones. They may participate in stress regulation processes by responding to hormones such as MeJA, ABA, and gibberellin (GA), thereby enhancing plants’ tolerance to adverse stresses. However, the specific mechanisms of action still require in-depth investigation. Previous studies have found that in *Pyrus bretschneideri*, the expression of *PbDIR4* increases significantly upon induction by SA, ABA, and MeJA, indicating that *PbDIR4* plays a crucial role in responses to biotic and abiotic stresses [24]. In addition, MYB transcription factor binding sites are present in the promoter regions of 95.5% (21) of *ClDIR* genes. Given that MYB transcription factors have been proven to play key regulatory roles in responses to abiotic stresses such as drought, salinity, and low temperature [45], it is speculated that MYB transcription factors may interact with *cis*-acting elements (such as MBS, MRE, W-box) in the promoters of *ClDIR* genes to coordinate the expression of *ClDIR* genes under hormone signaling and abiotic stress conditions, thereby enhancing plants’ environmental adaptability and regulating their growth and development processes.

### 3.6. Tissue-Specific and Fruit Development Expression Patterns of ClDIR Genes

The DIR gene family displayed distinct expression patterns across various tissues and developmental stages in watermelon. In this study, all 22 *ClDIR* genes were expressed in roots, stems, flowers, fruits, leaves, and tendrils, but with significant variations in expression levels. Notably, the majority of *ClDIR* genes showed higher expression in root tissues, a pattern consistent with tissue-specific expression of *SmDIR* genes in eggplant, where most *SmDIR* genes exhibited high root expression [29]. Previous studies have shown that DIR genes promote organ lignification, suggesting that the *ClDIR* genes are crucial for watermelon root development and may coordinate the regulation of other organ growth. Through comprehensive analysis of four transcriptome datasets, we investigated the gene expression of *ClDIR* genes during watermelon fruit development. Results showed that *ClDIR9*, *ClDIR12*, *ClDIR22*, *ClDIR20*, and *ClDIR11* maintained high expression throughout fruit development. These findings suggest that these five *ClDIR* genes may regulate fruit morphogenesis or exert critical functions in fruit quality formation.

### 3.7. Expression Profiles of ClDIR Genes Under Abiotic Stresses

To systematically investigate the functions of *ClDIR* genes in watermelon in response to abiotic stresses, we first analyzed the expression profiles of *ClDIR* genes using 7 sets of publicly available abiotic stress transcriptome datasets, including high temperature, drought, low light, salt, osmotic stress, nitrogen treatment, and cadmium treatment. Transcriptome analysis revealed that 63.64% of *ClDIR* genes exhibited significant differential expression under abiotic stresses, suggesting that this gene family is widely involved in the adaptive regulation of watermelon to abiotic adversities. Based on the differential expression results from transcriptome data, 6 key *ClDIR* genes (*ClDIR5*, *ClDIR8*, *ClDIR9*, *ClDIR12*, *ClDIR16*, and *ClDIR22*) with differential expression in abiotic stress responses were selected from 22 *ClDIR* genes, and their expression patterns under high-temperature, drought, and salt stresses were validated using qRT-PCR. The results showed that the expression trends of these six key genes were generally consistent with the transcriptome data. Among them, *ClDIR8*, *ClDIR9*, *ClDIR12*, and *ClDIR22* displayed highly consistent expression changes with the transcriptome data, with *ClDIR8* showing the highest consistency across all stress conditions. However, the expression characteristics of *ClDIR5* under high-temperature stress and *ClDIR16* under drought stress showed certain discrepancies with the transcriptome results, which may be attributed to differences in experimental conditions (e.g., stress intensity, duration) and plant materials. Nevertheless, the high consistency between qRT-PCR validation results and transcriptome data confirmed the reliability of the preliminary analysis. In addition, qRT-PCR analysis further revealed the response characteristics of these six genes to low-temperature stress. Compared with the 0 h control, the expression levels of all genes showed significant differences at most time points in both roots and leaves. Notably, *ClDIR8* exhibited extremely significant induced expression at all time points in leaves under low-temperature stress, with its expression level being approximately 30–40 fold higher than that of the control, suggesting that *ClDIR8* may play a central role in watermelon adaptation to low temperature. Combined transcriptome analysis and qRT-PCR validation indicated that members of the *ClDIR* genes play important regulatory roles in watermelon responses to multiple abiotic stresses. These findings are consistent with previous studies: *HpDIR16* and *HpDIR17* in *Herpetospermum pedunculosum* were significantly upregulated under salt stress, and silencing these two genes aggravated oxidative damage caused by salt stress, confirming their positive role in enhancing plant salt tolerance [46]. *PeDIR20* and *PeDIR43* in *Phyllostachys edulis* were significantly upregulated under drought and high-temperature stresses [26]. In maize, *ZmDIR11* was significantly upregulated in leaves under drought stress, and silencing *ZmDIR11* significantly reduced drought tolerance in maize seedlings, indicating its positive regulatory role in drought response [15]. Similarly, a study on sugarcane found that *ScDIR5*, *ScDIR7*, *ScDIR11*, and *ScDIR40* could enhance drought resistance in transgenic tobacco [14]. In this study, most *ClDIR* genes in watermelon were found to be induced by multiple abiotic stresses, further highlighting the critical regulatory roles of *ClDIR* genes in watermelon responses to abiotic stresses. Particularly, *ClDIR8*, which showed a robust response to abiotic stresses, may serve as a key candidate gene for watermelon adaptation to abiotic stresses, warranting in-depth investigation.

### 3.8. Role of ClDIR Genes in Biotic Stress Responses, with a Focus on ClDIR8

Previous studies have demonstrated that DIR family members play key regulatory roles in lignin biosynthesis and are involved in plant defense responses against microorganisms and insects [47,48,49]. Lignans, as widely distributed secondary metabolites in plants, are products of DIR-mediated reactions and exhibit significant antifungal activity. They enhance plant resistance to biotic stresses (e.g., pathogen infection) by inhibiting pathogen growth and spread [50]. For example, overexpression of *GhDIR1* in cotton (*Gossypium hirsutum*), *GmDIR22* in soybean (*Glycine max*), and *TaDIR13* in tobacco (*Nicotiana tabacum*) increases lignin accumulation and enhances resistance to *Verticillium dahliae*, *Phytophthora*, and *Pseudomonas syringae*, respectively [51,52,53]. Similarly, overexpression of *Fragaria vesca DIR13* in transgenic *Arabidopsis thaliana* lines increases lignin levels and activates genes in the phenylpropane and jasmonic acid signaling pathways, thereby enhancing resistance to *Colletotrichum higginsianum* [54]. Conversely, silencing pepper *CaDIR7* reduces root activity and increases plant susceptibility to *Phytophthora capsica* [28]. This study also analyzed the expression patterns of watermelon *ClDIR* genes under biotic stresses using nine sets of published biotic stress-related transcriptome datasets, including those for *Fusarium* wilt, cucumber green mottle mosaic virus, powdery mildew, squash vein yellowing virus, and root-knot nematodes. It was revealed that each of the 22 *ClDIR* genes exhibited differential expression under at least one biotic stress. Notably, *ClDIR8* showed significant differential expression across all nine biotic stress transcriptome datasets. These results indicate that *ClDIR* genes are widely involved in the transcriptional response of watermelon to diverse biotic stresses (e.g., fungal, viral, and nematode infections), and the unique expression pattern of *ClDIR8* suggests it may act as a key hub gene in watermelon’s response to biotic stresses. Future studies should validate the function of *ClDIR8* in watermelon or model crops using overexpression, gene editing, and other techniques to elucidate its molecular mechanism in regulating biotic stress responses, particularly whether it enhances resistance by promoting the synthesis of lignans and other disease-resistant metabolites.

### 3.9. Subcellular Localization of ClDIR8 and Identification of Interacting Proteins

Co-localization analysis with the peroxisomal marker protein SKL-mKate (red fluorescence) confirmed that ClDIR8 is localized to peroxisomes (Figure 10). Y2H assays showed that ClDIR8 can interact with the proteins Cla97C02G049920 and Cla97C08G152180. Further BiFC assays verified that Cla97C02G049920 and Cla97C08G152180 are genuine interacting proteins of ClDIR8. In the BiFC assays, punctate-distributed YFP fluorescence signals (yellow) were detected for both the ClDIR8-Cla97C02G049920 and ClDIR8-Cla97C08G152180 combinations. This fluorescence distribution pattern is consistent with the typical granular structural characteristics of peroxisomes and completely matches the peroxisomal localization result of ClDIR8 itself. Therefore, the BiFC assays not only confirmed that Cla97C02G049920 and Cla97C08G152180 are interacting proteins of ClDIR8, but also directly demonstrated, through the exhibited peroxisomal morphological characteristics, that ClDIR8 interacts with these two proteins inside peroxisomes.

### 3.10. Functional Implications of ClDIR8 Interactions with Peroxidase and Catalase, and Future Perspectives

This study is the first to experimentally confirm that ClDIR8 interacts with Cla97C02G049920 (encoding peroxidase) and Cla97C08G152180 (encoding catalase). Previous studies have reported that *Arabidopsis thaliana* AtDIR5/AtDIR6 can synergize with laccase to guide the directional coupling of coniferyl alcohol radicals, forming (−)-pinoresinol, an intermediate in the lignin pathway [34]. Similarly, in sesame, the initial stage of lignan synthesis was found to rely on laccase (LAC) and/or peroxidase (POD) to catalyze the oxidative coupling of two coniferyl alcohol molecules, with the stereoselective guidance of DIR proteins generating (+)-pinoresinol, a key precursor of lignans [55]. The above-mentioned studies indicate that the production of both (−)-pinoresinol and (+)-pinoresinol depends on the coordinated participation of DIR proteins and oxidases (LAC and/or POD). This study clarifies the interaction between ClDIR8 and the peroxidase-encoding gene Cla97C02G049920, and it is speculated that the two may guide the coupling of coniferyl alcohol radicals to generate pinoresinol with a specific configuration through a similar mechanism, thereby participating in the synthesis of lignans or lignin.

Notably, this study also revealed the interaction between ClDIR8 and the catalase-encoding gene Cla97C08G152180. Peroxidases can decompose various peroxides (ROOH) or aerobic organic compounds to generate H_2_O_2_ [56], while catalases can use H_2_O_2_ as a substrate to scavenge it and prevent cell damage [57]. Catalases play an important protective role when reactive oxygen species (ROS) are involved in biological reactions. As an essential substrate for peroxidases, the concentration of H_2_O_2_ may be mutually regulated by these two enzymes: when the activity of catalase is too high, it will lead to insufficient H_2_O_2_, inhibiting the peroxidase-mediated oxidation of lignin or lignan monomers; conversely, when the activity of catalase is low, the accumulation of H_2_O_2_ can enhance the catalytic efficiency of peroxidase. Given that the synthesis of both lignin and lignan requires oxidases (LAC and/or POD) to catalyze coniferyl alcohol to form a common precursor, the interaction between ClDIR8 and Cla97C08G152180 (catalase) may regulate the concentration of H_2_O_2_ and the scavenging of ROS at the reaction site, thereby affecting the synthesis efficiency of lignin or lignan.

Regarding the discovery that ClDIR8 interacts with peroxidase (POD) and catalase (CAT) inside peroxisomes, there are still many scientific questions awaiting in-depth investigation, for example, exploring whether ClDIR8, Cla97C02G049920, and Cla97C08G152180 might form a functional complex and deciphering the resistance regulatory mechanism of ClDIR8.

## 4. Materials and Methods

### 4.1. Identification of ClDIR Genes and Analysis of Physicochemical Properties of Their Encoded Proteins

To identify the members of the DIR gene family in watermelon, the genomic file of watermelon (97103_v2.5) was downloaded from the Cucurbit Genomics Database website (http://cucurbitgenomics.org/v2/, accessed on 15 March 2025) [58]. Then, the Hidden Markov Model (HMM) file (PF03018) corresponding to the DIR conserved domain was downloaded from the Pfam database (http://pfam.xfam.org/, accessed on 15 March 2025) [59]. Subsequently, the HMMER3.0 software was used to search for genes containing the DIR domain sequence in the watermelon protein database as candidate *ClDIR* genes [60]. Furthermore, by using the bam files from transcriptome sequencing of different watermelon tissues, the gene structure refinement of candidate *ClDIR* genes was performed using IGV-GSAman v0.6.83 software. Following refinement, the CDS and protein sequences of the candidate *ClDIR* genes were extracted. Online tools such as NCBI-CDD (https://www.ncbi.nlm.nih.gov/Structure/cdd/wrpsb.cgi, accessed on 15 March 2025) [61], Pfam (https://www.ebi.ac.uk/interpro/search/sequence/, accessed on 15 March 2025) [62], and SMART (https://smart.embl.de/, accessed on 15 March 2025) [63] were used to verify the candidate *ClDIR* genes. Genes containing the entire dirigent domain were selected as the final confirmed members of the *ClDIR* gene family. The Protein Parameter Calc (ProrParam-based) plugin in TBtools (v2.322) software [64] was utilized to analyze the physicochemical properties of DIR family proteins in watermelon, including coding sequence (CDS) length, amino acid number, molecular weight, isoelectric point (pI), instability index, aliphatic index, and the grand average of hydropathicity (GRAVY).

### 4.2. Phylogenetic Tree, Gene Structure, and Conserved Motif Analysis

The phylogenetic tree was constructed using MEGA-X (v10.2.6) software for 25 DIR proteins from *Arabidopsis thaliana* [27], 49 DIR proteins from rice (*Oryza sativa*) [19], 24 DIR proteins from pepper (*Capsicum annuum*) [28], 24 DIR proteins from eggplant (*Solanum melongena*) [29], 23 DIR proteins from cucumber (*Cucumis sativus*) [65], and 22 ClDIR proteins. The neighbor-joining (NJ) method was adopted with 1000 bootstrap replicates, using the Poisson model. The phylogenetic tree was visualized using the online software Evolview 2.0 (https://evolgenius.info/evolview-v2/, accessed on 16 March 2025) [66]. The exon, intron, and UTR sequences of each *ClDIR* gene were retrieved from the GFF3 file of the watermelon (97103_v2.5) genome. Conserved motifs of ClDIR proteins were identified using the MEME website (http://meme-suite.org/, accessed on 16 March 2025) [67], with parameters set as follows: 10 conserved motifs; optimal width of 6–100 amino acids. The Gene Structure View (Advanced) plugin in TBtools software was used for visual analysis of *ClDIR* gene exon/intron structures and ClDIR protein conserved motifs.

### 4.3. Synteny, Gene Duplication, and Selective Pressure Analysis of ClDIR Genes

To explore the collinearity of DIR family genes between watermelon and two model crops (*Arabidopsis thaliana* and rice), as well as between watermelon and two Cucurbitaceae species (cucumber and melon), the One Step MCScanX plugin in TBtools software was used. Collinearity analyses were conducted based on GFF3 and genome files, focusing on DIR family members in pairwise comparisons: watermelon vs. *A. thaliana*, watermelon vs. rice, watermelon vs. cucumber, and watermelon vs. melon. The Multiple Synteny Plot plugin in TBtools software was then employed to visualize interspecific collinear gene pairs.

Within the watermelon DIR gene family, tandemly and segmentally duplicated genes were first identified using MCScanX (v1.0.0) software [68]. Tandem duplication events were further verified via the One Step MCScanX function in TBtools. Interspecific collinearity relationships were visualized using the Advanced Circos plugin in TBtools software.

Selective pressure analysis was performed using KaKs_calculator (v2.0) [69] to calculate non-synonymous substitution rates (Ka), synonymous substitution rates (Ks), and Ka/Ks ratios for duplicated *ClDIR* gene pairs.

### 4.4. Cis-Acting Element Analysis of ClDIR Gene Promoters

The PlantCARE database (http://bioinformatics.psb.ugent.be/webtools/plantcare/html/, accessed on 17 March 2025) [70] was used for bioinformatics prediction of *cis*-acting elements in the 2000 bp promoter region upstream of *ClDIR* genes. Raw data obtained were systematically organized, classified, and statistically analyzed using Microsoft Excel 2019. The heatmap plugin in TBtools software was then employed to visualize the *cis*-acting elements of *ClDIR* genes. Furthermore, Origin 2021 software was used to generate a stacked bar chart depicting the number of *cis*-acting elements in each *ClDIR* gene for intuitive analysis.

### 4.5. Reanalysis of Watermelon Transcriptome Sequencing Data

Watermelon transcriptome data were retrieved and downloaded from the NCBI SRA database (https://www.ncbi.nlm.nih.gov/sra, accessed on 4 August 2024) and National Genomics Data Center of China (https://ngdc.cncb.ac.cn/, accessed on 5 August 2024). The downloaded SRA-format data were converted to Fastq format using fasterq-dump (v2.11.0) (https://github.com/ncbi/sra-tools/wiki/HowTo:-fasterq-dump, accessed on 10 August 2024), and data quality was assessed via a quality report generated by FastQC (v0.11.9) [71]. Low-quality sequences were filtered using Trimmomatic (v0.39) to obtain high-quality reads [72]. Filtered Fastq files were aligned to the watermelon (97103_v2.5) genome using STAR (v2.7.11b), generating SAM-format files [73]. These were converted to BAM format and sorted using SAMtools (v1.18) [74]. Transcript expression levels were calculated with StringTie (v2.2.1) [75], and differentially expressed genes (DEGs) were identified using DESeq2 (v1.40.2) by inputting gene count matrices, thus completing the DEG analysis [76].

### 4.6. Analysis of ClDIR Gene Expression Patterns in Different Tissues and Under Abiotic and Biotic Stresses

Transcriptome sequencing data for different watermelon tissues and stress conditions (abiotic/biotic) were retrieved from the NCBI SRA database (https://www.ncbi.nlm.nih.gov/sra, accessed on 4 August 2024) and National Genomics Data Center of China (https://ngdc.cncb.ac.cn/, accessed on 5 August 2024) (Table 3). Using watermelon genomic information (97103_v2.5), the transcriptome data were reanalyzed following the pipeline described above. TBtools software was then used to generate expression heatmaps of *ClDIR* genes in different tissues and during abiotic and biotic stress responses. Additionally, differentially expressed *ClDIR* genes were screened based on DEG results from each treatment group.

### 4.7. Plant Materials and Stress Treatments

An inbred watermelon line W-22-13 was used to analyze the expression patterns of *ClDIR* genes in response to abiotic stresses. Seeds of W-22-13 were sterilized with 55 °C water for 10 min, soaked for 8 h, and germinated in an incubator at 28 °C. Post-germination, seeds were planted in a growing medium and cultured in a growth chamber under controlled conditions: 27 °C/16 h (day), 25 °C/8 h (night), 25,000 lux light intensity, and 60% humidity. At the two-leaf stage, seedlings with uniform growth were selected for abiotic stress treatments. The experiment included four groups—high-temperature treatment (45 °C), low-temperature treatment (4 °C), salt stress (root application of 500 mmol·L^−1^ NaCl), and drought stress (root application of 20% PEG6000)—with 24 seedlings per group. For temperature stresses, seedlings were transferred to growth chambers set at target temperatures. For salt/drought treatments, 10 mL of each solution was evenly applied to the root system to ensure full root exposure to stressors. Root and leaf samples were collected at 0, 6, 12, and 24 h post-treatment. At each time point, samples from two seedlings were pooled to form one biological replicate, with three replicates per time point. All samples were immediately frozen in liquid nitrogen and stored at −80 °C for subsequent analysis.

### 4.8. Total RNA Extraction and qRT-PCR Analysis

Total RNA was extracted from samples using the FastPure Universal Plant Total RNA Isolation Kit (Vazyme Biotech Co., Ltd., Nanjing, China). RNA concentration was measured with a NanDrop 2000c Spectrophotometer (Thermo Scientific, Waltham, MA, USA), and its integrity was verified by 1% agarose gel electrophoresis. cDNA was synthesized according to the manufacturer’s protocol using the HiScript^®^ III RT SuperMix for qPCR (+gDNA wiper) (Vazyme Biotech Co., Ltd., Nanjing, China). Quantitative real-time polymerase chain reaction (qRT-PCR) was performed on a ViiA7 Real-Time PCR System (Applied Biosystems, Waltham, MA, USA) with ChamQ Universal SYBR qPCR Master Mix (Vazyme Biotech Co., Ltd., Nanjing, China) to determine the relative expression levels of *ClDIR* genes under four abiotic stress conditions. The *β-Actin* gene was used as an internal reference gene for normalization in qRT-PCR analysis. The relative expression levels were calculated following the 2^−ΔΔCT^ method [91]. Primer sequences are listed in Table S4.

### 4.9. Subcellular Localization Analysis of ClDIR8 Protein

The coding sequence of *ClDIR8* (excluding the stop codon) was cloned into the plant expression vector pAN580-eGFP, harboring the enhanced green fluorescent protein (eGFP) reporter gene under the control of the constitutive 35S Cauliflower Mosaic Virus (CaMV) promoter. The resultant constructs, 35S::*ClDIR8*-eGFP (experimental), 35S::eGFP (empty vector control), and 35S::SKL-mKate (peroxisomal marker) [92], were introduced into *Arabidopsis thaliana* mesophyll protoplasts using a polyethylene glycol (PEG)-mediated transient expression system [93]. Fluorescence signals were observed under a Nikon C2-ER laser confocal microscope. Primer sequences are listed in Table S4.

### 4.10. Y2H Library Screening

The full-length coding sequence of the *ClDIR8* gene was cloned into the bait vector pGBKT7 and transformed into the yeast strain Y2HGold. Prior to library screening, self-activation and toxicity of the ClDIR8 bait protein were validated on SD/-Trp/-Leu/-His/-Ade and SD/-Trp selective media. A cDNA library derived from watermelon root and leaf tissues was constructed in the prey vector pGADT7 using Gateway^®^ recombination technology. The pGBKT7-*ClDIR8* bait vector and the prey cDNA library were co-transformed into Y2HGold cells. Transformants were screened on SD/-Trp/-Leu/-His/-Ade plates supplemented with 20 μg/mL X-α-Gal at 30 °C for 3–5 days. Blue-colored positive clones were subjected to colony PCR, Sanger sequencing, and BLAST analysis to identify interacting proteins.

### 4.11. Y2H Validation and BiFC Assays

For Y2H validation, candidate prey plasmids were co-transformed with the bait plasmid into Y2HGold cells, and interactions were confirmed on SD/-Trp/-Leu/-His/-Ade plates and by β-galactosidase filter lift assays. For BiFC assays, the bait and prey genes (*ClDIR8* and interacting partners) were cloned into pCAMBIA1300-35S-N and pCAMBIA1300-35S-C vectors, respectively, to fuse them with the N-terminal (YFP^N^) and C-terminal (YFP^C^) fragments of YFP. Recombinant constructs were co-transformed into *Nicotiana benthamiana* leaves via *Agrobacterium tumefaciens* GV3101-mediated infiltration. Following 48 h incubation at 25 °C, YFP fluorescence was visualized using a Zeiss LSM880 confocal laser scanning microscope with excitation at 514 nm and emission at 527–535 nm. Negative controls included empty vector transformations and co-expression of non-fused YFP^N^ and YFP^C^ fragments to exclude self-complementation artifacts. Primer sequences are listed in Table S4.

## 5. Conclusions

In this study, we identified 22 *ClDIR* genes in watermelon. By integrating analyses of physicochemical properties, chromosomal localization, gene structure, phylogeny, collinearity, and expression patterns, we gained insights into the evolution and expression characteristics of *ClDIR* genes under diverse environments. Our results revealed that *ClDIR8* was involved in responses to all 16 stress conditions in transcriptome analysis, and its responsiveness was validated by qRT-PCR under high-temperature, low-temperature, salt, and drought stresses. Additionally, the *ClDIR8* protein was localized to peroxisomes, and its interacting proteins (Cla97C02G049920 and Cla97C08G152180) were identified using yeast two-hybrid (Y2H) and bimolecular fluorescence complementation (BiFC) assays. Overall, these findings lay a foundation for further functional studies of *ClDIR* genes and provide potential candidate genes for breeding stress-resistant watermelon.

## Figures and Tables

**Table 1:** The physiochemical characteristics of DIR family genes in watermelon.

Gene Name	Gene ID	CDS Size (bp)	Number of Amino Acids (aa)	Molecular Weight (kDa)	Theoretical pI	Instability Index	Aliphatic Index	Grand Average of Hydropathicity
*ClDIR1*	*Cla97C01G006790*	561	186	20.36	8.89	29.32	90.70	0.118
*ClDIR2*	*Cla97C02G028400*	549	182	19.88	6.71	24.00	96.98	0.332
*ClDIR3*	*Cla97C02G035380*	525	174	18.44	9.64	9.42	99.77	0.272
*ClDIR4*	*Cla97C02G037290*	576	191	21.00	9.10	30.27	86.75	−0.127
*ClDIR5*	*Cla97C02G037300*	555	184	19.80	9.19	30.59	92.66	0.147
*ClDIR6*	*Cla97C02G039150*	1191	396	41.46	4.36	43.18	79.34	−0.140
*ClDIR7*	*Cla97C02G039160*	738	245	27.50	9.63	33.34	68.86	−0.329
*ClDIR8*	*Cla97C02G050020*	570	189	20.91	9.39	28.23	94.87	0.145
*ClDIR9*	*Cla97C02G050030*	564	187	20.45	10.04	29.08	101.18	0.167
*ClDIR10*	*Cla97C03G055500*	1200	399	43.45	6.14	52.00	77.74	−0.337
*ClDIR11*	*Cla97C05G084350*	753	250	25.62	5.00	36.26	88.60	0.224
*ClDIR12*	*Cla97C05G088170*	549	182	20.06	5.41	38.82	93.63	0.116
*ClDIR13*	*Cla97C06G110310*	603	200	22.53	6.51	42.14	89.80	−0.007
*ClDIR14*	*Cla97C06G124580*	564	187	20.62	8.47	44.13	86.52	0.016
*ClDIR15*	*Cla97C06G124950*	552	183	20.04	7.89	36.89	92.19	0.236
*ClDIR16*	*Cla97C07G134890*	573	190	21.15	8.38	22.75	83.74	0.127
*ClDIR17*	*Cla97C07G134900*	579	192	21.16	7.74	44.41	86.35	0.169
*ClDIR18*	*Cla97C07G134920*	558	185	20.59	8.95	32.29	84.43	0.039
*ClDIR19*	*Cla97C09G181680*	951	316	32.50	4.95	34.91	80.22	0.002
*ClDIR20*	*Cla97C09G184270*	546	181	20.24	9.69	38.17	85.75	−0.223
*ClDIR21*	*Cla97C10G184750*	582	193	21.15	7.34	27.10	94.87	0.172
*ClDIR22*	*Cla97C10G200650*	579	192	20.89	9.69	54.36	93.44	0.223

**Table 2:** Ka/Ks analysis for the duplicated *ClDIR* paralogous gene pairs in watermelon.

Duplicated Gene Pairs	Ka	Ks	Ka/Ks	Duplication	Selection Pattern
*ClDIR4*/*ClDIR5*	0.48	1.33	0.36	Tandem	Purifying selection
*ClDIR8*/*ClDIR9*	0.29	1.09	0.26	Tandem	Purifying selection
*ClDIR16*/*ClDIR17*	0.19	0.55	0.35	Tandem	Purifying selection
*ClDIR2/ClDIR3*	0.43	2.06	0.21	Segmental	Purifying selection
*ClDIR6*/*ClDIR7*	1.06	0.83	1.28	Tandem	Positive selection

**Table 3:** Transcriptome datasets of watermelon for analyzing *ClDIR* gene expression patterns in diverse tissues and under stresses.

Project	No.	Experiment	Accession Number	Sampled Tissue	Reference
Tissue-specific expression	1	Different tissues	PRJNA1031825	Root, stem, leaf, male flower, female flower, fruit, tendril	-
	2	Fruit development	PRJNA407607	Fruit flesh	[77]
	3	Fruit development	PRJNA718123	Fruit flesh	-
	4	Fruit development	PRJNA703434	Fruit flesh	[78]
	5	Fruit development	PRJNA520808	Fruit flesh	[79]
Abiotic stresses	6	High temperature	PRJNA504354	Ovule	-
	7	Drought	PRJNA604984	Leaf	[80]
	8	Low light	PRJNA602124	Fruit flesh	[81]
	9	Salt	PRJNA609260	Leaf	[82]
	10	Osmotic stress	PRJNA770012	Leaf	[83]
	11	Nitrogen treatment	PRJNA422970	Leaf, root	[84]
	12	Cadmium stress	PRJNA1079538	Leaf	-
Biotic stresses	13	*Fusarium* wilt	PRJNA641525	Root	[85]
	14	*Fusarium* wilt	PRJNA794199	Root	[86]
	15	*Fusarium* wilt	PRJNA973274	Root	-
	16	*Fusarium* wilt	PRJNA929333	Leaf	[87]
	17	*Fusarium* wilt	PRJNA783543	Root	[88]
	18	Cucumber green mottle mosaic virus	PRJNA534308	Leaf	-
	19	Powdery mildew	PRJNA881394	Leaf	[89]
	20	Squash vein yellowing virus	PRJNA1086032	Leaf	-
	21	Root-knot nematodes	PRJCA001078	Leaf	[90]

## Data Availability

All associated data are presented in the article.

## Supplementary Materials

The following supporting information can be downloaded at: https://www.mdpi.com/article/10.3390/ijms26167730/s1.

## Abbreviations

The following abbreviations are used in this manuscript:

DIR	Dirigent proteins
BiFC	Bimolecular fluorescence complementation
Y2H	Yeast two-hybrid
GRAVY	Grand average of hydropathicity
pI	Theoretical isoelectric points
Ka	Non-synonymous substitution rates
Ks	Synonymous substitution rates
ABA	Abscisic acid
SA	Salicylic acid
MeJA	Methyl jasmonate
GA	Gibberellin
DAP	Days after pollination
dpi	Day post inoculation
YFP	Yellow fluorescent protein
CDS	Coding sequence
NJ	Neighbor-joining method
DEGs	Differentially expressed genes
qRT-PCR	Quantitative real-time polymerase chain reaction
eGFP	Enhanced green fluorescent protein
