# Supplementary figures and images for: The DIR Gene Family in Watermelon: Evolution, Stress Expression Profiles, and Functional Exploration of ClDIR8
Source: Int J Mol Sci. 2025 Aug 10;26(16):7730. doi: 10.3390/ijms26167730 (PMC12386893; doi:10.3390/ijms26167730)

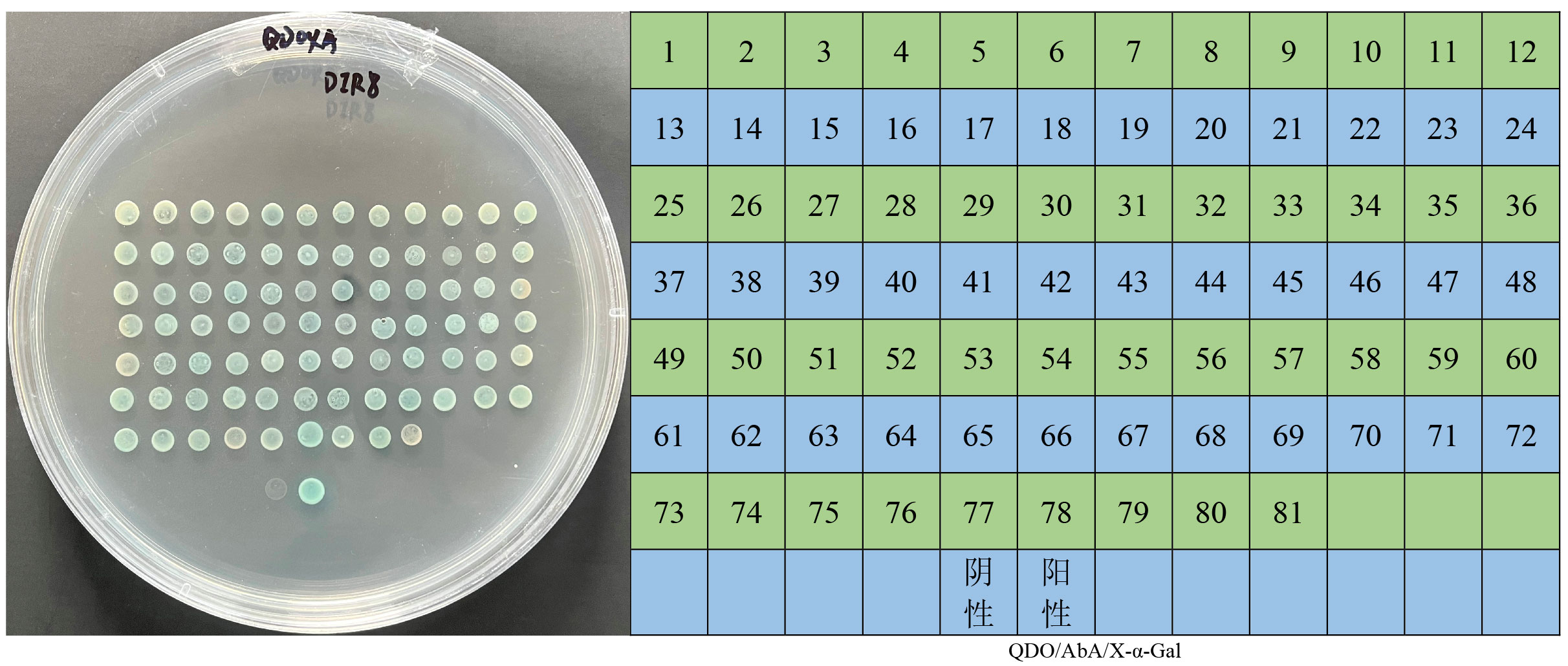

Supplement: Supplementary file 1 [file ijms-26-07730-s001.zip › Figure S1-The initial screening of interacting proteins of ClDIR8.jpg]
